# HDAC9 Silencing Exerts Neuroprotection Against Ischemic Brain Injury *via* miR-20a-Dependent Downregulation of NeuroD1

**DOI:** 10.3389/fncel.2020.544285

**Published:** 2021-01-11

**Authors:** Liangjun Zhong, Jinxiang Yan, Haitao Li, Lei Meng

**Affiliations:** ^1^Department of Neurosurgery, Pingyin County People’s Hospital, Jinan, China; ^2^Department of Neurosurgery, Ningyang No. 1 People’s Hospital, Tai’an, China; ^3^Department of Neurology, Qihe County People’s Hospital, Dezhou, China; ^4^Department of Neurosurgery, Shandong Provincial Hospital, Jinan, China

**Keywords:** HDAC9, microRNA-20a, NeuroD1, ischemic brain injury, OGD cell models 3

## Abstract

Cerebral stroke is an acute cerebrovascular disease that is a leading cause of death and disability worldwide. Stroke includes ischemic stroke and hemorrhagic strokes, of which the incidence of ischemic stroke accounts for 60–70% of the total number of strokes. Existing preclinical evidence suggests that inhibitors of histone deacetylases (HDACs) are a promising therapeutic intervention for stroke. In this study, the purpose was to investigate the possible effect of HDAC9 on ischemic brain injury, with the underlying mechanism related to microRNA-20a (miR-20a)/neurogenic differentiation 1 (NeuroD1) explored. The expression of HDAC9 was first detected in the constructed middle cerebral artery occlusion (MCAO)-provoked mouse model and oxygen-glucose deprivation (OGD)-induced cell model. Next, primary neuronal apoptosis, expression of apoptosis-related factors (Bax, cleaved caspase3 and bcl-2), LDH leakage rate, as well as the release of inflammatory factors (TNF-α, IL-1β, and IL-6) were evaluated by assays of TUNEL, Western blot, and ELISA. The relationships among HDAC9, miR-20a, and NeuroD1 were validated by *in silico* analysis and ChIP assay. HDAC9 was highly-expressed in MCAO mice and OGD-stimulated cells. Silencing of HDAC9 inhibited neuronal apoptosis and inflammatory factor release *in vitro*. HDAC9 downregulated miR-20a by enriching in its promoter region, while silencing of HDCA9 promoted miR-20a expression. miR-20a targeted Neurod1 and down-regulated its expression. Silencing of HDAC9 diminished OGD-induced neuronal apoptosis and inflammatory factor release *in vitro* as well as ischemic brain injury *in vivo* by regulating the miR-20a/NeuroD1 signaling. Overall, our study revealed that HDAC9 silencing could retard ischemic brain injury through the miR-20a/Neurod1 signaling.

## Introduction

Stroke is the commonest cause of death in China and the second reason for death globally, thus bringing high morbidity, disability, and high cost of treatment, which is often classified into ischemic stroke and hemorrhagic stroke (Pandian et al., 2018; Yao et al., 2019). The incidence of ischemic stroke is higher than that of hemorrhagic stroke, accounting for 60–70% of the total number of strokes (Guzik and Bushnell, 2017). Although current treatment approaches consist of non-pharmacological, pharmacological, and surgical approaches (such as endovascular therapy, mechanical thrombectomy, and tirofiban; Derex and Cho, 2017; Almekhlafi et al., 2020; Zhou et al., 2020), there is a need for optimization of biochemical marker analyses, and hopefully an extension to more specific and sensitive methods to depict disease processes and to diagnose the disease at an early stage, while also providing potential new therapeutic channels.

Recent studies have found that epigenetic mechanisms, including DNA methylation, histone deacetylation, and microRNAs (miRNAs), are essential for the pathogenesis of ischemic cerebral infarction (Picascia et al., 2015), among which the role of histone deacetylases (HDACs) has been studied in stroke and other neurological diseases (Gomazkov, 2015). In animal cerebral infarction models, it has also been demonstrated the neuroprotection of a variety of HDAC inhibitors, by effectively inhibiting the secondary damage due to inflammatory response and apoptotic pathways, thereby reducing the scope of stroke and improving the resultant neurological deficits (Kim et al., 2007; Langley et al., 2009; Aune et al., 2015; Ganai et al., 2016; Park and Sohrabji, 2016). However, the mechanisms of HDACs in stroke outcomes are unclear. The HDACs family has multiple subtypes, and the roles and mechanisms of different subtypes are different (Aune et al., 2015). It is worth having an in-depth understanding of the role of each subtype in cerebral infarction, which can further reveal the pathophysiology of ischemic cerebral infarction. Nowadays, the role and mechanisms of some subtypes in the pathophysiology of ischemic cerebral infarction are being explored. For example, the expression of HDAC3/6 is increased in cerebral infarction models, and its knockout or inhibitor attenuates the neurological damage after infarction (Chen et al., 2012). The expression of another subtype of HDAC, HDAC4, is elevated in middle cerebral artery (MCA) occlusion (MCAO) and oxygen-glucose deprivation (OGD) models, and it is involved in the reconstruction of synapses and nerve repair after the injury (Kassis et al., 2015; Yuan et al., 2016). The possible involvement of histone deacetylase 9 (HDAC9) in cerebral infarction has not been thoroughly studied, although recent genomic correlation studies have found that HDAC9 variant genes may be closely related to the outcome of aortic stroke (Azghandi et al., 2015; Qingxu et al., 2016), suggesting that HDAC9 may also be involved in the pathophysiology of ischemic cerebral infarction.

In this study, we observed the expression of HDAC9 after cerebral infarction, thus exploring its relationship with the occurrence of cerebral infarction, to further understand the mechanisms of HDACs in ischemic stroke. We aimed to identify key targets and molecular interventions to improve the prevention, treatment, and prognosis of cerebral infarction.

## Materials and Methods

### Ethics Statement

The experimental procedures involving the animals were all employed with the approval of the Institutional Animal Use Committee of Shandong Provincial Hospital. Extensive efforts were made with the attempt to guarantee the discomfort of the used animals was minimal.

### Construction of MCAO-Induced Mouse Ischemic Brain Injury Models

Thirty-five 8–10 weeks C57BL/6 mice (weighing from 22 g to 25 g) from the Experimental Animal Center of Shandong Provincial Hospital were used to induce transient MCAO. After being fasted for 8 h, the mice were subjected to anesthesia by intraperitoneal injections of pentobarbital. The mouse model of MCAO was performed as described previously, with minor modifications (Chi et al., 2014; Yang et al., 2017; Park et al., 2019; Zhou et al., 2019). Briefly, mice were deeply anesthetized with an intraperitoneal injection (i.p.) of pentobarbital sodium (60 mg/kg). Blunt dissection was performed, under a stereomicroscope (Stemi 2000, Carl Zeiss, Dresden, Germany), to expose the left common carotid artery (CCA), left external carotid artery (ECA), and left internal common carotid artery (ICA). The CCA artery was temporarily occluded using a suture, while the ECA artery was permanently sutured as distally as possible, and another was sutured distally to the bifurcation. This was followed by a small incision in the CCA artery between permanent and temporary sutures, a 5–0 surgical nylon filament with a round tip (0.23 mm in diameter) was inserted into the internal carotid artery about 12 mm beyond the carotid bifurcation, thereby occluding the origin of the MCA. After 1-h MCAO, the mice were allowed to recover for 24 h by removing the suture. In the sham group, all procedures were identical except for the insertion of an intraluminal filament. During the surgical procedures, the core body temperature was maintained at 36.5–37.0°C using a heating pad and the respiratory rate was monitored continuously. All the mice had a neurologic deficit assessment at 24 h of reperfusion, and the brains were removed quickly for later analysis. Finally, we obtained 24 MCAO mice, giving the successful rate of modeling was 88.89%. These MCAO mice were subsequently classified into three treatment groups (*n* = 8): MCAO + short hairpin RNA negative control (sh-NC) + overexpression (oe)-NC, MCAO + sh-HDAC9 + oe-NC, and MCAO + sh-HDAC9 + oe-Neurod1. An osmotic pump was used to continuously pump sh-HDAC9 and oe-Neurod1 into the lateral ventricle, as well as the corresponding control lentivirus into the control group, once a day for 7 days. Intracerebral stereotactic injection was conducted as follows: after intraperitoneal injection of 3% pentobarbital sodium for anesthesia, the mice were subjected to intraventricular injection of 4 μl, 1 μl/min of each lentivirus (titer of 2 × 10^8^ IFU/ml) with the use of The Mouse Brain Atlas (Second Edition, George Paxinos, Keither B J Franklin), stereotaxic apparatus (Kopf, Tujunga CA, USA), and stepper motorized microsyringe (Hamilton, Bonaduz, Switzerland), and then kept in place for 5–10 min before withdrawal. One hour after the MCAO model was induced, the virus was injected as previously described (Li et al., 2019). Briefly, after anesthesia using 5% pentobarbital sodium, the right common, external, and internal carotid arteries (ICAs) of the mice were surgically exposed, followed by ligation of the common carotid arteries. Following this, a 7/0 silicone-coated monofilament nylon suture (0.22–0.23 mm in diameter) with a round tip was gently introduced into the ICAs through the ECA and advanced 10 ± 0.5 mm until the round tip reached the branch to the right MCA when a slight resistance was felt. Laser Doppler flowmetry (PeriFlux 5000, Järfälla, Sweden) was applied for validation of the successful occlusion. Two hours after the occlusion, the suture was carefully withdrawn to restore blood flow. After recovery and observation of their neurobehaviors, the mice were euthanized and the cerebral infarction volume was measured.

### Primary Hippocampal Neuron Culture

Embryos of 12–14-day C57BL/6 mice were used to prepare primary hippocampal neurons (Al Rahim and Hossain, 2013). The isolated mouse embryos were separated into hippocampal tissues, which were then sliced into sections. After 5-min of trypsinization (0.125%) and 5-min of centrifugation at 500 rpm, the neurons were resuspended using 10% fetal bovine serum- (GIBCO, Frederick, MD, USA) supplemented DMEM. Next, the cells (2.5 × 10^5^ cells/cm^2^) were inoculated into the poly-L-lysine- (Sigma-Aldrich, St.Louis, MO, USA) coated cultured dishes for 4 h. Subsequently, the cells were subjected to a neurobasal medium containing 2% B27 and 0.5 mM L-GlutaMa-I (Gibco, Frederick, MD, USA) at 37°C in 5% CO_2_ for 7 days for the subsequent experiments.

### Immunofluorescence

After 12-min of fixation using 4% formaldehyde and 4% sucrose, the neurons were permeabilized by 0.25% Triton X-100 for 5 min at 17 days. Then, 10% bovine serum albumin (BSA) in phosphate-buffered saline (PBS) for 30 min was used to block the neurons, which were subsequently subjected to overnight incubation at 4°C with the following primary antibodies: rabbit anti synapsin I (1:2,000), mouse immunoglobulin G (IgG)1 anti drebrin 1 (Clone M2F6; 1:8, hybridoma supernatant), chicken polyclonal IgY anti MAP2 (1:5,000), and mouse IgG2a anti-Tau-1 (Clone PC1C6; 1:2,000, recognizes dephosphorylated tau). After that, the neurons were further incubated for 1 h at 37°C with the species-or isotype-specific secondary antibodies as follows: Alexa 488-conjugated anti-mouse IgG2a (1:500), Alexa 568-conjugated anti-rabbit (1:500), Alexa 647-conjugated anti-mouse IgG1 (1:500), and AMCA-conjugated anti-chicken IgY (donkey IgG, 1:200).

### Neurological Evaluation

The neurological deficits of MCAO mice were evaluated by two observers blindly and scored according to the Longa score method at 6, 12, and 24 h after surgery, and the average of the two scores was obtained. 0 point indicated normal, no neurological deficits; 1 point indicated left forelimb extension disorder, mild neurological deficits; 2 points indicated turning left while walking, moderate neurological deficit; 3 points indicated body collapse to the left, severe neurological deficit; and 4 points indicated an inability to spontaneously move, unconscious.

### 2,3,5-Triphenyl Tetrazolium Chloride (TTC) Staining

After neurobehavioral testing, the mouse brains were collected and frozen sections were prepared. The brains of mice in each group were dissected, frozen, and cut into coronal sections of approximately 1.5 mm in thickness. Then stained with 2% 2,3,5-triphenyl tetrazolium chloride (TTC; Sigma–Aldrich, St.Louis, MO, USA) for 10 min at 37°C and fixed in 10% PFA overnight. In this staining procedure, TTC reacts with physiological succinate dehydrogenase and turns red, while ischemic tissue stays pale due to decreased dehydrogenase activity. Representative images were taken using a digital camera. The infarction volume was blindly analyzed as percentages of the contralateral structures.

### Isolation and Quantification of the RNAs

Following the measurement of the cerebral infarction volume, a reverse transcription-quantitative polymerase chain reaction (RT-qPCR) was conducted. In short, the isolation of total RNAs from the whole brain tissues or cells in different groups was employed using the TRIzol reagent (15596026, Invitrogen, Carlsbad, CA, USA). After that, the complementary DNA (cDNA) of miRNA was synthesized using a miRNA First Strand cDNA Synthesis (B532451-0020, Shanghai Biotech, Shanghai, China), while that of non-miRNA using the cDNA reverse transcription kit (K1622, Beijing Ya and a Biotechnology Company Limited, Beijing, China Tailing Reaction). Next, the synthesized cDNAs were subject to RT-qPCR with the use of a Fast SYBR Green PCR kit (Applied Biosystems, Foster City, CA, USA), which were subsequently subjected to an ABI 7500 instrument (Applied Biosystems, Foster City, CA, USA), with each reaction run in triplicate. All used primer sequences are presented in Table 1. Finally, with normalized to U6 and glyceraldehyde-3-phosphate dehydrogenase (GAPDH), the 2^−△△CT^ method was utilized for calculation on the fold changes.

**Table 1:** Primer sequences for reverse transcription-quantitative polymerase chain reaction (RT-qPCR).

Target	Sequence (5′-3′)
miR-20a	Forward: GGGGTAAAGTGCTTATAGTGC
	Reverse: CAGTGCGTGTCGTGGAGT
U6	Forward: ACAGAGAAGATTAGCATGGCC
	Reverse: GACCAATTCTCGATTTGTGCG
HDAC9	Forward: GTCCCTGCCCAATATCAC
	Reverse: GCTGTTCGGTTTGCCCTC
GAPDH	Forward: AACGGATTTGGTCGTATTGGG
	Reverse: TCGCTCCTGGAAGATGGTGAT

### Western Blot Analysis

After the measurement of cerebral infarction volume, Western blot analysis was conducted as following steps. After trypsinization, an enhanced radioimmunoprecipitation assay (RIPA) lysis buffer (Boster Biological Technology Company Limited, Wuhan, China) containing protease inhibitor was applied to lyse the cells. Then, the measurement of protein concentration was employed with the use of bicinchoninic acid (BCA) protein assay kit (Boster Biological Technology Company Limited, Wuhan, China). The separated proteins by freshly-prepared sodium dodecyl sulfate-polyacrylamide gel electrophoresis (SDS-PAGE) were transferred onto polyvinylidene fluoride membranes, which were probed at 4°C overnight with the following-mentioned primary antibodies (Abcam, Cambridge, UK): HDAC9 (ab59718, 1:1,000), cleaved caspase3 (ab49822, 1:500), Bcl-2-associated X protein (Bax; ab32503, 1:500), B-cell lymphoma 2 (bcl-2; ab185002, 1:500), Neurod1 (ab205300, 1:1,000), and GAPDH (ab181602, 1:5,000). The next day, after being re-probed at 37°C with goat anti-rabbit IgG (ab205718, 1:10,000, Abcam, Cambridge, UK) for 1 h, the membranes were subjected to enhanced chemiluminescence reagent and captured under a SmartView Pro 2000 (UVCI-2100, Major Science, Saratoga, CA, USA) gel imaging system for immunoblot visualization. With GAPDH as the normalization, the target protein bands were quantified by employing the Quantity One software.

### OGD-Induced Cell Model Establishment

The mouse primary hippocampal neurons were cultured for 7 days, whereupon the medium was replaced with glucose-free Dulbecco’s modified Eagle’s medium (DMEM). The cells were placed in an anaerobic culture system (GeneScience AG30 Anaerobic workstation system, USA) of 95% N2/5% CO_2_ at 37°C and so cultured for 3 h of hypoxia treatment. Next, the cells were removed from the hypoxic culture dish, and the glucose-free DMEM was replaced with the neuron culture medium. The cells were then placed back in the incubator at 37°C, with 5% CO_2_ and normal oxygen concentration for 6–24 h. At the same time, the cells in the normal group received no treatment. OGD-treated cells were then grouped into sh-NC, sh-HDAC9, sh-NC + NC inhibitor, sh-HDAC9 + NC inhibitor, sh-HDAC9 + miR-20a inhibitor, mimic NC + oe-NC, miR-20a mimic + oe-NC, miR-20a mimic + oe-Neurod1, sh-NC + oe-NC, sh-HDAC9 + oe-NC, and sh-HDAC9 + oe-Neurod1 groups. In these groups, lentiviral vectors carrying LV5-GFP (for gene overexpression, addgene, #25,999), and pSIH1-H1-copGFP (for gene silencing, System Biosciences, LV601B-1) were used to infect primary cortical neurons. 293T cells were employed for lentiviral packaging. In brief, 293T cells were cultured in Roswell Park Memorial Institute (RPMI)-1640 medium containing 10% fetal bovine serum and then sub-cultured every other day. The CaCl2 method was used for the transfection of lentivirus packaged plasmids, and the virus (1 × 10^8^ TU/ml) was added to neurons for infection, and then OGD treatment was conducted. Meanwhile, the plasmids of mimic NC, miR-20a mimic, NC inhibitor, and miR-20a inhibitor were used to transfect cells using the Lipofectamine 2000 reagent (Invitrogen Inc., Carlsbad, CA, USA) following the manufacturer’s instructions.

### Terminal Deoxynucleotidyl Transferase-Mediated dUTP-Biotin Nick End Labeling (TUNEL) Assay

The primary neurons at the logarithmic growth phase in different treatment groups and the control group were collected and their apoptosis was detected using the *in situ* Cell Death Detection Kit (Cat. No. 11684795910, Roche, Basel, Switzerland), which could label the nicked TdT-mediated dUTP. The kit was used according to the manufacturer’s instructions with small modulations. We seeded cells onto poly-L-lysine coated glass coverslips and fixed these cells in 4% paraformaldehyde (PFA) for 60 min when they have grown to 60% confluence. Equilibration buffer was added to the coverslips before incubating them with nucleotide mix and rTdT enzyme at 37°C for 60 min. After stopping the reaction with a 23 saline–sodium citrate buffer, the cells were mounted with an anti-fade mounting medium, followed by fluorescence microscopically observation for the fluorescence intensity.

### Determination of Lactate Dehydrogenase (LDH) Leakage Rate

The LDH was measured using a CytoTox96 non-radioactisve cytotoxicity detection kit (Promega, Madison, WI, USA). In brief, the neurons were inoculated into a coated 96-well plate at a density of 1 × 10^5^−1 × 10^6^ cells/well, and then incubated at 37°C, in the 5% CO_2_ incubator. After hypoxia treatment for 1 h, the cells were removed from the hypoxic culture dish and the glucose-free DMEM was replaced with the neuron medium. The hippocampal neurons were cultured in a 5% CO_2_ incubator at 37°C at normal oxygen concentration for 6–24 h. Next, the cells were added with 15 μl lysis (9% v/v) Triton^®^ X-100 in water) and incubated for 45 min in a 37°C incubator. Then, 50 μl of the cell supernatant was added to another 50 μl 96-well plate preloaded with substrate mixture and reacted for 30 min at room temperature in the dark. Next, 50 μl stop solution was added to each well to stop the reaction. Finally, the absorbance value was measured using a microplate reader at 490 nm. LDH = absorbance value _cell culture fluid_/(absorbance value _cell culture fluid_ + absorbance value _cell homogenate_) × 100%.

### Enzyme-Linked Immunosorbent Assay (ELISA)

Brain tissues were extracted, from which the infarct area or the corresponding area of the control group were isolated, and then homogenized and centrifuged at 3,500 rotations/min for 10 min. After 3 h of hypoxia treatment, the cells were removed from the hypoxic culture dish, and the glucose-free DMEM was replaced with the neuron medium. The hippocampal neurons were cultured in a 5% CO_2_ incubator at 37°C at normal oxygen concentration for 6–24 h before collection of the supernatant. Then the contents of inflammatory factors including tumor necrosis factor-α (TNF-α; JLC3924), interleukin (IL)-1β (JLC3580), and IL-6 (JLC3601) in tissue homogenates or the cell supernatant were determined by ELISA according to the kit instructions (Shanghai Jingkang Biological Engineering Company Limited, Shanghai, China).

### Chromatin Immunoprecipitation (ChIP)

The experiment was conducted with the use of a ChIP kit (Millipore, Bedford, MA, USA). Neurons in each group were subjected to trypsinization and fixation in 1% formaldehyde for DNA and protein cross-linking. Next, the complex was randomly fractured by ultrasonic treatment (120 w, 2 s on, 5 s off for each time) to produce fragment, followed by centrifugation at 13,000 rpm at 4°C. Following this step, the collected supernatant was separated into three tubes, which were supplemented with positive control antibodies RNA polymerase II or NC antibody normal human IgG and anti-HDAC9 (1:100, ab59718, Abcam Cambridge, UK). After overnight incubation at 4°C, the endogenous DNA-protein complex was precipitated with the use of protein Agarose/Sepharose. With the supernatant aspirated and discarded post transient centrifugation, the non-specific complexes were washed to de-crosslink the complex overnight at 65°C. After that, the phenol/chloroform extraction purification was used to recover the DNA fragments, followed by an examination of the miR-20a promoter expression by RT-qPCR.

### Dual-Luciferase Reporter Assay

293T cells were seeded in a six-well plate at a density of 2 × 10^5^ cells/well. After successful transfection and adherence, the cells were cultured for 48 h and collected. Next, the luciferase activity of Neurod1 in cells was detected according to the method provided by the Genecopoeia’s dual-luciferase detection kit (D0010, Solibao Technology Company Limited, Beijing, China). The Glomax 20/20 luminometer fluorescence detector (E5311, Shanxi Zhongmei Biotechnology Company Limited, Shaanxi, China) was used to detect the fluorescence intensity.

### Hematoxylin-Eosin (HE) Staining

Mice anesthetized using isoflurane were subjected to thoracotomy, perfused with normal saline at 4°C followed by 4% paraformaldehyde. After overnight post-fixation in 10% neutral formaldehyde solution, the brain tissues were subsequently paraffin-embedded and sectioned. Next, xylene dewaxing and gradient alcohol hydration were performed for the sections, which were subjected to hematoxylin staining for 8 min and eosin staining for 2 min, respectively. Following routine dehydration, clearing, and resin-covering, the morphological changes of cells were microscopically observed with high magnification.

### Statistical Analysis

SPSS 21.0 statistical software (IBM Corp., Armonk, NY, USA) was used for data processing. Measurement data were presented as mean ± standard deviation from at least three independent experiments. Data obeying normal distribution and homogeneity of variance between two groups were compared using unpaired *t*-test and comparisons among multiple groups were assessed by one-way analysis of variance (ANOVA), followed by Tukey’s tests with corrections for multiple comparisons. *p* < 0.05 was considered to be statistically significant.

## Results

### HDAC9 Expresses Highly in MCAO-Induced Mouse and OGD-Provoked Cell Models of Ischemic Brain Injury

**Figure 1:**
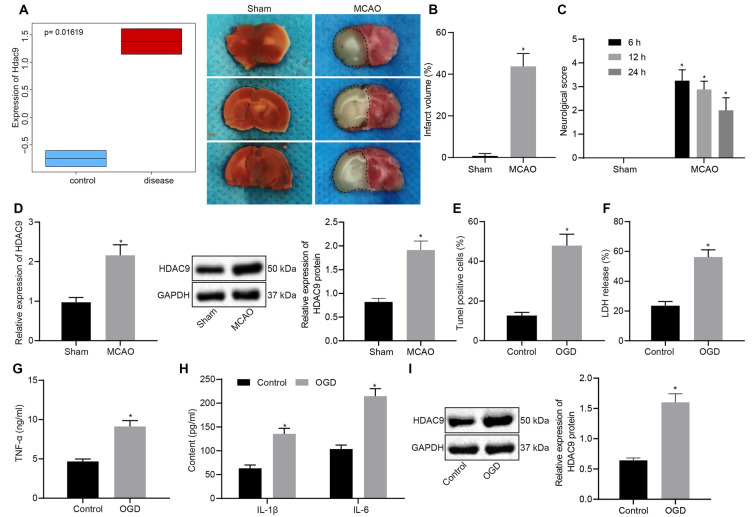
Histone deacetylases (HDAC9) is highly expressed in middle cerebral artery occlusion (MCAO)-induced mouse and oxygen-glucose deprivation (OGD)-provoked cell models of ischemic brain injury. **(A)** A box plot depicting HDAC9 expression in GSE28201 dataset (*p* = 0.01619). **(B)** 2,3,5-triphenyl tetrazolium chloride (TTC) staining of mouse cerebral tissues of sham-operated and MCAO mice; the black dotted line represents the ischemic injury. **(C)** Neurobehavioral score at 6, 12, 24 h after MCAO induction. **(D)** HDAC9 expression in the cerebral tissues of sham-operated and MCAO mice detected by reverse transcription-quantitative polymerase chain reaction (RT-qPCR), normalized to glyceraldehyde-3-phosphate dehydrogenase (GAPDH). **(E)** Western blot analysis of HDAC9 protein in cerebral tissues of sham-operated and MCAO mice, normalized to GAPDH. **(F)** Number of TUNEL-positive primary neurons in cerebral tissues of sham-operated and MCAO mice. **(G)** Lactate dehydrogenase (LDH) leakage rate determination in control and OGD-induced neurons. **(H)** Expression levels of inflammatory factors (TNF-α, IL-1β, and IL-6) in control and OGD-induced neurons detected by ELISA. **(I)** Western blot analysis of HDAC9 protein in control and OGD-induced neurons, normalized to GAPDH. *p < 0.05 vs. the sham or control group. Measurement data (mean ± standard deviation) between the two groups were compared using an unpaired *t*-test. *N* = 8.

We first differentially analyzed the gene expression on a cerebral ischemia-related expression dataset GSE28201 retrieved from the Gene Expression Omnibus (GEO) database and then plotted a box plot illustrating the most distinct differentially expressed genes. As shown in Figure 1A, HDAC9 was among the upregulated gene. Besides, we checked the expression of HDAC9 in different tissues and cells in the bioGPS website and found that HDAC9 was highly expressed in dendritic cells, macrophages, microglia, neuro2a1 cells, hippocampal neurons, and olfactory bulb, especially in the olfactory bulb and hippocampal tissues (Supplementary Figure 1) To understand the involvement of HDCA9 in ischemic brain injury, a mouse model of ischemic brain injury was established. TTC staining observed an increased cerebral infarct area in the MCAO mice (Figure 1B). As neurobehavioral Longa scores at 6, 12, and 24 h post MCAO presented, relative to the sham-operated mice, the MCAO mice had higher neurological scores (Figure 1C), indicating the successful construction of MCAO-induced mouse models.

The analyses by RT-qPCR and Western blot implicated that, relative to the sham-operated mice, the MCAO mice exhibited elevated HDAC9 expression (Figures 1D,E). To understand the role of HDAC9 in the OGD-induced cell model, we performed a series of assays *in vitro* and found that, OGD treatment led to an increase in cell apoptosis, LDH leakage rate, and the expression of inflammatory factors (TNF-α, IL-1β, IL-6; Figures 1F–H, and Supplementary Figure 2A), indicating that the OGD cell model was successfully constructed. The Western blot results demonstrated that HDAC9 protein expression was increased in the cells following OGD induction (Figure 1I). These experimental data indicated an elevation in the HDAC9 expression in the mouse and cell models.

### Silencing of HDAC9 Impeded OGD-Induced Neuron Apoptosis and Production of Inflammatory Factors *In vitro*

**Figure 2:**
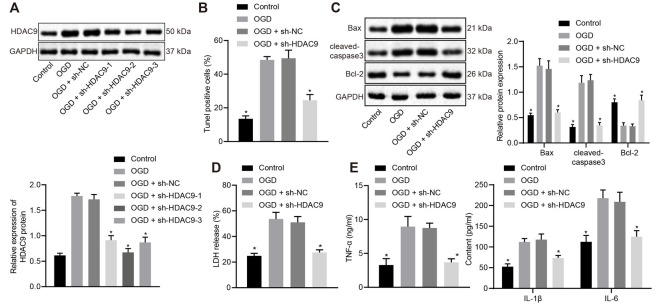
Silencing of HDAC9 inhibits OGD-induced neuronal apoptosis and the expression of inflammatory factors *in vitro*. **(A)** Knockdown efficiency of HDAC9 confirmed by Western blot analysis in OGD-induced neurons treated with sh-HDAC9-1, sh-HDAC9-2 and sh-HDAC9-3, normalized to GAPDH. **(B)** Number of TUNEL-positive primary OGD-induced neurons treated with sh-HDAC9. **(C)** Western blot analysis of cell apoptosis-related proteins (Bax, cleaved caspase3 and bcl-2) in OGD-induced neurons treated with sh-HDAC9, normalized to GAPDH. **(D)** LDH leakage rate determination in OGD-induced neurons treated with sh-HDAC9. **(E)** Expression of inflammatory factors (TNF-α, IL-1β, and IL-6) detected by ELISA in OGD-induced neurons treated with sh-HDAC9. *p < 0.05 vs. the OGD + sh-NC group. Measurement data (mean ± standard deviation) between two groups were compared using unpaired *t-test* and those among multiple groups were assessed by one-way ANOVA, followed by Tukey’s *post hoc* tests. *n* = 3.

To understand the effect of HDAC9 on the apoptosis and inflammatory response of OGD-treated neurons, we silenced HDAC9 and detected the expression of HDAC9 by Western blot analysis. The obtained data revealed that, relative to the sh-NC group, the sh-HDAC9-1, sh-HDAC9-2, and sh-HDAC9-3 groups, all showed reduced HDAC9 expression, with the best silencing effect in the sh-HDAC9-2 group (Figure 2A), which was thus selected for subsequent experiments. As TUNEL and Western blot analysis suggested, relative to the OGD + sh-NC group, cell apoptosis, as well as the expressions of Bax and cleaved caspase3, were diminished in the OGD + sh-HDAC9 group, but the expression of bcl-2 was augmented (Figures 2B,C and Supplementary Figure 2B). Measurement of the LDH leakage rate showed a tendency for reduction in the OGD + sh-HDAC9 group relative to the OGD + sh-NC group (Figure 2D), while the same trend was identified in the expression of TNF-α, IL-1β, and IL-6 by ELISA between these two groups (Figure 2E). The above results indicated that silencing of HDAC9 inhibited OGD-induced neuronal apoptosis and the release of inflammatory factors *in vitro*.

### HDAC9 Downregulated miR-20a Expression *In vitro*

**Figure 3:**
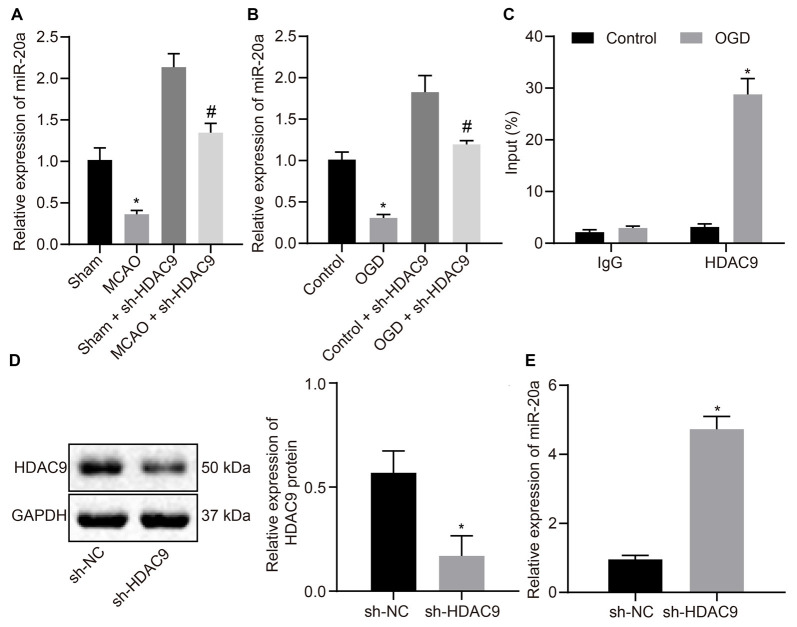
HDAC9 negatively regulates miR-20a expression *in vitro*. **(A)** miR-20a expression in brain tissues of MCAO mice or MCAO mice treated with sh-HDAC9 detected by RT-qPCR (*n* = 8), normalized to U6. **(B)** miR-20a expression in OGD-treated neurons or OGD-treated neurons treated with sh-HDAC9 detected by RT-qPCR, normalized to U6. **(C)** HDAC9 enrichment in the miR-20a promoter region detected by ChIP. **(D)** Western blot analysis of HDAC9 protein in OGD-treated neurons transfected with sh-HDAC9, normalized to GAPDH. **(E)** miR-20a expression in OGD-treated neurons transfected with sh-HDAC9 detected by RT-qPCR, normalized to U6. *p < 0.05 vs. the sham, control, or sh-NC group. Measurement data (mean ± standard deviation) between the two groups were compared using an unpaired *t*-test. *n* = 3. ^#^*p* < 0.05 vs. the sham + sh-HDAC9 group or the control + sh-HDAC9 group.

To verify whether HDAC9 can regulate the expression of miR-20a, we used RT-qPCR to detect the expression of miR-20a in MCAO mouse and OGD-induced cell models. The results illustrated that miR-20a expression was much higher in the MCAO-sh-HDAC9 and OGD-sh-HDAC9 groups than the MCAO and OGD groups, respectively. Compared to the sham and control groups, miR-20a expression was decreased in the MCAO-sh-HDAC9 and OGD-sh-HDAC9 groups, respectively (Figures 3A,B). ChIP revealed increased enrichment of HDAC9 in the miR-20a promoter region in the OGD group in comparison with the control group (Figure 3C). Also, a higher protein expression of HDAC9 was found in the sh-HDAC9 group than the sh-NC group (Figure 3D). The expression of miR-20a detected by RT-qPCR presented an upward trend in the sh-HDAC9 group in comparison to the sh-NC group (Figure 3E). Therefore, HDAC9 inhibited the expression of miR-20a by enriching in its promoter region, and HDCA9 silencing promoted miR-20a expression *in vitro*.

### Silencing of HDAC9 Inhibited OGD-Induced Neuron Apoptosis and Inflammatory Factor Production *via* miR-20a Promotion *In vitro*

**Figure 4:**
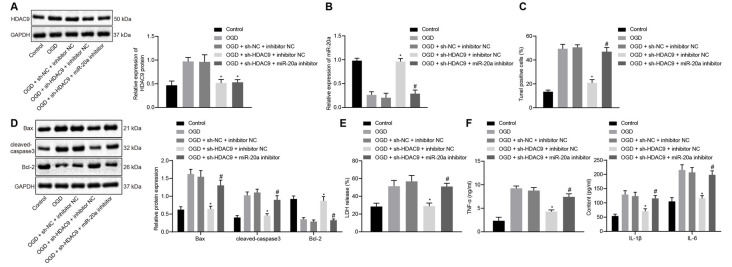
Silencing of HDAC9 promotes miR-20a expression and inhibits OGD-induced neuronal apoptosis *in vitro*. **(A)** Western blot analysis of HDAC9 protein in OGD-treated neurons treated with sh-HDAC9 or in combination with miR-20a inhibitor, normalized to GAPDH. **(B)** Expression of miR-20a detected by RT-qPCR in OGD-treated neurons treated with sh-HDAC9 or in combination with miR-20a inhibitor, normalized to U6. **(C)** Number of TUNEL-positive primary OGD-treated neurons treated with sh-HDAC9 or in combination with miR-20a inhibitor. **(D)** Western blot analysis of cell apoptosis-related proteins (Bax, cleaved caspase3, bcl-2) in OGD-treated neurons treated with sh-HDAC9 or in combination with miR-20a inhibitor, normalized to GAPDH. **(E)** LDH leakage rate determination in OGD-treated neurons treated with sh-HDAC9 or in combination with miR-20a inhibitor. **(F)** Expression of inflammatory factors (TNF-α, IL-1β, and IL-6) in OGD-treated neurons treated with sh-HDAC9 or in combination with miR-20a inhibitor measured by ELISA. *p < 0.05 vs. the OGD + sh-NC + inhibitor NC group, and ^#^*p* < 0.05 vs. the OGD + sh-HDAC9 + inhibitor NC group. Measurement data (mean ± standard deviation) among multiple groups were assessed by one-way ANOVA, followed by Tukey’s *post hoc* tests. *n* = 3.

To understand the effect of HDAC9 and miR-20a on the apoptosis and inflammatory response of OGD-treated neurons, we silenced HDAC9 and inhibited the expression of miR-20a. The data demonstrated that relative to the OGD + sh-NC + inhibitor NC group, HDAC9 expression was reduced in the OGD + sh-HDAC9 + inhibitor NC group and the OGD + sh-HDAC9 + miR-20a inhibitor group (Figure 4A). The expression of miR-20a detected by RT-qPCR displayed an increase in the OGD + sh-HDAC9 + inhibitor NC group vs. the OGD + sh-NC + inhibitor NC group. As compared to the OGD + sh-HDAC9 + inhibitor NC group with the OGD + sh-HDAC9 + miR-20a inhibitor group, a lower expression of miR-20a was observed in the latter group (Figure 4B).

Figures 4C,D and Supplementary Figure 2C illustrated the results of TUNEL and Western blot analysis that, as compared with the OGD + sh-NC + inhibitor NC group, cell apoptosis together with the expression of Bax and cleaved caspase3 was diminished in the OGD + sh-HDAC9 + inhibitor NC group, while the expression of bcl-2 was increased. In contrast, both sh-HDAC9 and miR-20a inhibitor transfection resulted in an enhancement in cell apoptosis and the expression of Bax and cleaved caspase3, yet a decline in bcl-2 expression (Figures 4C,D and Supplementary Figure 2C).

When compared with the group of OGD + sh-NC + inhibitor NC, the LDH leakage rate as well as the expression of TNF-α, IL-1β, and IL-6 showed a decline in the group of OGD + sh-HDAC9 + inhibitor NC, while the group of OGD + sh-HDAC9 + miR-20a inhibitor exhibited an increased tendency relative to the group of OGD + sh-HDAC9 + inhibitor NC (Figures 4E,F). The above results indicated that silencing of HDAC9 promoted miR-20a expression and inhibited OGD-induced neuronal apoptosis *in vitro*.

### miR-20a Suppressed OGD-Induced Apoptosis and Production of Inflammatory Factors by Targeting NeuroD1 *In vitro*

**Figure 5:**
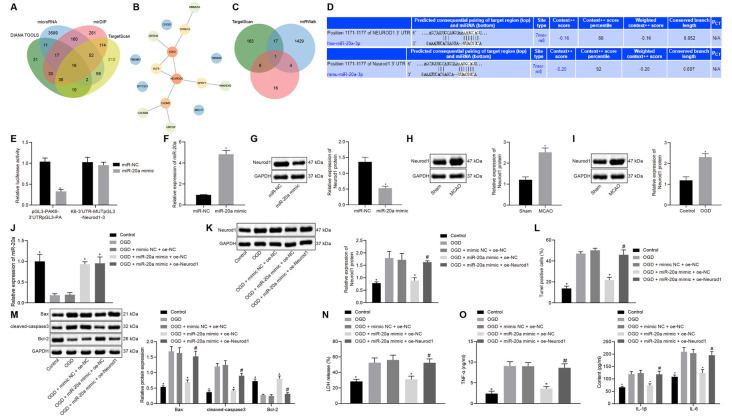
miR-20a impedes OGD-induced neuronal apoptosis by targeting Neurod1. **(A)** Venn diagram of the downstream genes of human miR-20a predicted by the DIANA TOOLS, microRNA, mirDIP, and TargetScan databases. **(B)** PPI network diagram of 16 intersecting genes constructed by String. Deeper red in the circle where genes were localized reflects a higher core degree, and conversely deeper blue reflects a lower core degree. **(C)** Venn map of downstream genes of mouse miR-20apredicted by TargetScan, miRWalk and DIANA TOOL databases. **(D)** Binding sites between miR-20a and Neurod1 in human and mouse predicted by the TargetScan website. **(E)** Binding of miR-20a to Neurod1 verified using dual-luciferase reporter assay. **(F)** Expression of miR-20a in cells transfected with miR-20a mimic detected by RT-qPCR, normalized to U6. **(G)** Western blot analysis of Neurod1 protein in cells transfected with miR-20a mimic, normalized to GAPDH. **(H)** Western blot analysis of Neurod1 protein in brain tissues of MCAO mice (*n* = 8). **(I)** Western blot analysis of Neurod1 protein in control and OGD-treated neurons, normalized to GAPDH. **(J)** miR-20a expression detected by RT-qPCR in OGD-treated neurons transfected with miR-20a mimic or in combination with oe-Neurod1, normalized to U6. **(K)** Western blot analysis of HDAC9 protein in OGD-treated neurons transfected with miR-20a mimic or in combination with oe-Neurod1, normalized to GAPDH. **(L)** Number of TUNEL-positive primary OGD-treated neurons transfected with miR-20a mimic or in combination with oe-Neurod1. **(M)** Western blot analysis of cell apoptosis-related proteins (Bax, cleaved caspase3, and bcl-2) in OGD-treated neurons transfected with miR-20a mimic or in combination with oe-Neurod1, normalized to GAPDH. **(N)** LDH leakage rate determination in OGD-treated neurons transfected with miR-20a mimic or in combination with oe-Neurod1. **(O)** Expression of inflammatory factors (TNF-α, IL-1β, and IL-6) in OGD-treated neurons transfected with miR-20a mimic or in combination with oe-Neurod1 detected by ELISA. Panels **(E–I)**: *p < 0.05 vs. the sham, OGD or mimic NC group; panels **(J–O)**: *p < 0.05 vs. the OGD + mimic NC + oe-NC group; and ^#^*p* < 0.05 vs. the OGD + miR-20a mimic + oe-Neurod1 group. Measurement data (mean ± standard deviation) between two groups were compared using unpaired *t-test* and those among multiple groups were assessed by one-way ANOVA, followed by Tukey’s *post hoc* tests. *n* = 3.

Using the databases DIANA TOOLS, microRNA, mirDIP, and TargetScan, we obtained 155, 3906, 708, and 601 downstream genes of human miR-20a respectively, intersection analysis revealed 16 intersecting genes with differential expression (Figure 5A). The PPI network of these 16 genes was constructed by String, which showed that EZH2 and Neurod1 were the genes with the highest core degree through Cytoscape beautification and calculation of the core degree (Figure 5B; Table 2). TargetScan, miRWalk, and DIANA TOOL were used to predict the downstream genes of mouse miR-20a, which revealed that the most critical gene was Neurod1 (Figure 5C). Furthermore, the binding relationship between human and mouse miR-20a and Neurod1 was predicted from TargetScan (Figure 5D), which was confirmed by dual-luciferase experiments. Compared with the NC group, the luciferase activity of the Neurod1 wild type was reduced in the miR-20a mimic group, but that of Neurod1 mutant showed no obvious changes (Figure 5E), indicating the presence of a binding relationship between miR-20a and Neurod1.

**Table 2:** Core degree of input genes in the PPI network.

Rank	Gene	Degree
1	EZH2	4
1	NEUROD1	4
3	CADM2	3
4	DYRK1A	2
4	KLF9	2
4	SPRY1	2
7	ZBTB38	1
7	UBE2W	1
7	ANKRD50	1
7	RMND5A	1
7	CACNB4	1
12	CPEB3	0
12	TMEM67	0
12	SPTY2D1	0
12	MED17	0
12	TMSB4X	0

RT-qPCR results revealed increased miR-20a expression in the miR-20a mimic group vs. the NC group (Figure 5F). Relative to the NC group, the protein expression of Neurod1 was reduced in the miR-20a mimic group (Figure 5G). Western blot analysis also demonstrated a highly-expressed Neurod1 in ischemic brain injury mouse models and OGD cell models (Figures 5H,I).

OGD-treated cells were subsequently transfected with overexpressed miR-20a and Neurod1 for subsequent experiments. Compared with the OGD + mimic NC + oe-NC group, the expression of miR-20a was increased in the OGD + miR-20a mimic + oe-NC group (Figure 5J). Compared with the OGD + mimic NC + oe-NC group, the expression of Neurod1 was reduced in the OGD + miR-20a mimic + oe-NC group. Compared with the OGD + miR-20a mimic + oe-NC group, the expression of Neurod1 was increased in the OGD + miR-20a mimic + oe-Neurod1 group (Figure 5K).

Additionally, as compared to the OGD + mimic NC + oe-NC group, cell apoptosis along with the expression of Bax and cleaved caspase3 was attenuated, while bcl-2 expression was increased in the OGD + miR-20a mimic + oe-NC group. Compared with the OGD + miR-20a mimic + oe-NC group, the OGD + miR-20a mimic + oe-Neurod1 group showed increased cell apoptosis and expression of Bax and cleaved caspase3, yet reduced bcl-2 expression (Figures 5L,M and Supplementary Figure 2D).

Further, the experimental data presented that in comparison with the OGD + mimic NC + oe-NC group, the LDH leakage rate was reduced in the OGD + miR-20a mimic + oe-NC group but was higher in the OGD + miR-20a mimic + oe-Neurod1 group than in the OGD + miR-20a mimic + oe-NC group (Figure 5N).

ELISA showed that, compared with the OGD + mimic NC + oe-NC group, the expression of TNF-α, IL-1β, and IL-6 was reduced in the OGD + miR-20a mimic + oe-NC group but was much lower in the OGD + miR-20a mimic + oe-Neurod1 group than in the OGD + miR-20a mimic + oe-NC group (Figure 5O). These experimental data indicated that miR-20a down-regulated Neurod1 expression, thus inhibiting the OGD-induced neuronal apoptosis and the expression of inflammatory factors *in vitro*.

### Silencing of HDAC9 Inhibited OGD-Induced Neuron Apoptosis and Inflammatory Factor Production Through the miR-20a/NeuroD1 Axis *In vitro*

**Figure 6:**
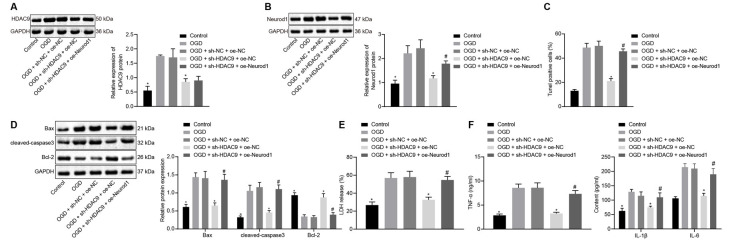
Silencing ofHDAC9 suppresses OGD-induced neuronal apoptosis through regulation of the miR-20a/Neurod1 signaling *in vitro*. **(A)** Western blot analysis of HDAC9 protein in OGD-treated neurons transfected with sh-HDAC9 or in combination with oe-Neurod1, normalized to GAPDH. **(B)** Western blot analysis of Neurod1 protein in OGD-treated cells transfected with sh-HDAC9 or in combination with oe-Neurod1, normalized to GAPDH. **(C)** Number of TUNEL-positive primary OGD-treated neurons transfected with sh-HDAC9 or in combination with oe-Neurod1. **(D)** Western blot analysis of cell apoptosis-related proteins (Bax, cleaved caspase3 and bcl-2) in OGD-treated neurons transfected with sh-HDAC9 or in combination with oe-Neurod1, normalized to GAPDH. **(E)** Determination of LDH leakage rate in OGD-treated neurons transfected with sh-HDAC9 or in combination with oe-Neurod1. **(F)** Expressions of inflammatory factors (TNF-α, IL-1β, and IL-6) in OGD-treated neurons transfected with sh-HDAC9 or in combination with oe-Neurod1 detected by ELISA. *p < 0.05 vs. the OGD + sh-NC + oe-NC group, and ^#^*p* < 0.05 vs. the OGD + sh-HDAC9 + oe-NC group. Measurement data (mean ± standard deviation) among multiple groups were assessed by one-way ANOVA, followed by Tukey’s *post hoc* tests. *n* = 3.

Next, we shifted our attention to understand the effect of HDAC9 and Neurod1 on the apoptosis and inflammatory response of OGD-treated neurons. Compared with the OGD + sh-NC + oe-NC group, the protein expression of HDAC9 and Neurod1 was reduced in the OGD + sh-HDAC9 + oe-NC group (Figures 6A,B). However, the expression of Neurod1 was increased in the OGD + sh-HDAC9 + oe-Neurod1 compared with that in the OGD + sh-HDAC9 + oe-NC group (Figure 6B).

The results of TUNEL and Western blot analysis showed that, compared with the OGD + sh-NC + oe-NC group, cell apoptosis, as well as the expression of Bax and cleaved caspase3, was reduced, while the expression of bcl-2 was increased in the OGD + sh-HDAC9 + oe-Neurod1 group. There were opposite changes in the OGD + sh-HDAC9 + oe-Neurod1 group when compared with the OGD + sh-HDAC9 + oe-NC group (Figures 6C,D and Supplementary Figure 2E). Compared with the OGD + sh-NC + oe-NC group, the LDH leakage rate was reduced in the OGD + sh-HDAC9 + oe-NC group, whereas it was higher in the OGD + sh-HDAC9 + oe-Neurod1 group than that in the OGD + sh-HDAC9 + oe-NC group (Figure 6E).

ELISA results demonstrated reduced expression of TNF-α, IL-1β, and IL-6 in the OGD + sh-HDAC9 + oe-NC group compared with the OGD + sh-NC + oe-NC group, but the elevated expression in the OGD + sh-HDAC9 + oe-Neurod1 group compared with the OGD + sh-HDAC9 + oe-NC group (Figure 6F). Taken together, silencing of HDAC9 inhibited OGD-induced neuronal apoptosis and inflammatory response through regulation of the miR-20a/Neurod1 axis *in vitro*.

### Silencing of HDAC9 Inhibited Ischemic Brain Injury Through the miR-20a/NeuroD1 Axis *In vivo*

**Figure 7:**
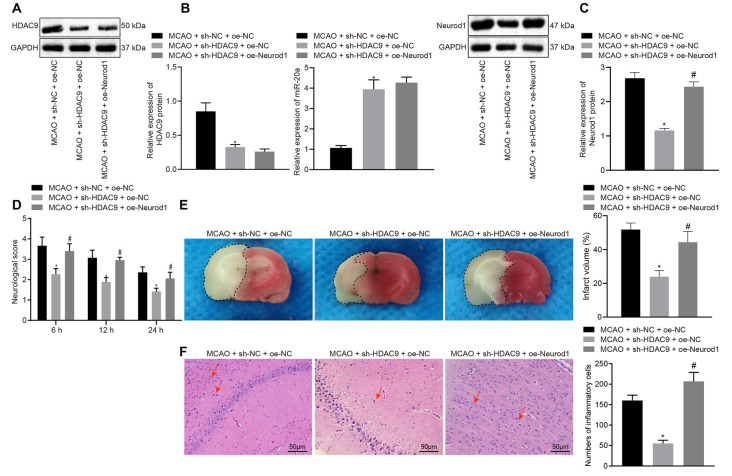
Silencing of HDAC9 hinders ischemic brain injury through regulation of the miR-20a/Neurod1 axis *in vivo*. **(A)** Western blot analysis of HDAC9 protein in brain tissues of MCAO mice treated with sh-HDAC9 or in combination with oe-Neurod1, normalized to GAPDH. **(B)** The expression of miR-20a in brain tissues of MCAO mice treated with sh-HDAC9 or in combination with oe-Neurod1 detected by RT-qPCR, normalized to U6. **(C)** Western blot analysis of Neurod1 protein in brain tissues of MCAO mice treated with sh-HDAC9 or in combination with oe-Neurod1, normalized to GAPDH. **(D)** Neurological function of MCAO mice treated with sh-HDAC9 or in combination with oe-Neurod1 evaluated by Longa score. **(E)** TTC staining of brain tissues of MCAO mice treated with sh-HDAC9 or in combination with oe-Neurod1; the black dotted line represents the ischemic injury. **(F)** Hematoxylin-eosin (HE) staining of brain tissues of MCAO mice treated with sh-HDAC9 or in combination with oe-Neurod1 (left panel). Quantification data for the total numbers of inflammatory cells in each section (right panel). Red arrow refers to infiltrated inflammatory cells. *p < 0.05 vs. the MCAO + sh-NC + oe-NC group, and ^#^*p* < 0.05 vs. the MCAO + sh-HDAC9 + oe-NC group. Measurement data (mean ± standard deviation) among multiple groups were assessed by one-way ANOVA, followed by Tukey’s *post hoc* tests. *N* = 8 for mice upon each treatment.

To further verify the above mechanisms *in vivo*, we constructed an MCAO model of ischemic brain injury and then injected sh-HDAC9 and oe-Neurod1 into the injured brain. Western blot assay implicated a reduced HDAC9 expression in the MCAO + sh-HDAC9 + oe-NC group compared with the MCAO + sh-NC + oe-NC group (Figure 7A). As RT-qPCR revealed, relative to the group of MCAO + sh-NC + oe-NC, the expression of miR-20a was increased in the group of MCAO + sh-HDAC9 + oe-NC (Figure 7B). Also, the expression of Neurod1 was reduced in the MCAO + sh-HDAC9 + oe-NC group compared with the MCAO + sh-NC + oe-NC group but was increased in the MCAO + sh-HDAC9 + oe-Neurod1 group in comparison with the MCAO + sh-HDAC9 + oe-NC group (Figure 7C).

Furthermore, the mouse neurological deficit score was lower in the MCAO + sh-HDAC9 + oe-NC group than that in the MCAO + sh-NC + oe-NC group but was higher in the MCAO + sh-HDAC9 + oe-Neurod1 group compared to the MCAO + sh-HDAC9 + oe-NC group (Figure 7D). TTC staining showed that the cerebral infarct size and extent of brain injury were reduced in the MCAO + sh-HDAC9 + oe-NC group compared with the MCAO + sh-NC + oe-NC group, but were increased in the MCAO + sh-HDAC9 + oe-Neurod1 group in relation to the MCAO + sh-HDAC9 + oe-NC group (Figure 7E). HE staining analysis revealed that infiltrated inflammatory cells were increased in the MCAO + sh-NC + oe-NC group, but opposite results were noted in the MCAO + sh-HDAC9 + oe-NC group. Moreover, quantification for the total numbers of inflammatory cells in each section showed that reduced inflammatory cells in the MCAO + sh-HDAC9 + oe-NC group relative to the MCAO + sh-NC + oe-NC group, while an increasing trend was observed in the MCAO + sh-HDAC9 + oe-Neurod1 group compared with the MCAO + sh-HDAC9 + oe-NC group. However, in the MCAO + sh-HDAC9 + oe-Neurod1 group, there were more infiltrated inflammatory cells, indicating that HDAC9 and NeuroD1 may play an important role in this brain injury model. Red arrows in Figure 7F indicate infiltrated inflammatory cells. The above results indicated that HDAC9 silencing inhibited ischemic brain injury through the miR-20a/Neurod1 axis *in vivo*.

## Discussion

HDACs have been identified to be essential for epigenetically regulating the transcription, while HDAC inhibitors are used as the neuroprotective agents in a variety of settings (Park and Sohrabji, 2016). Also, a strong association of HDAC9 with large vessel ischemic stroke has been well-documented (Bellenguez et al., 2012). In this current investigation, the inhibition of HDAC9 was found to restrain neuron apoptosis and production of inflammatory factors by regulating the miR-20a/Neurod1 axis, thus underscoring the therapeutic potential of molecules inhibiting HDAC9 for ischemic brain injury therapy.

HDAC9, a chromatin-modifying enzyme, is widely expressed in brain tissues and functions importantly in the development (Sugo and Yamamoto, 2016) and maintenance of the nervous system (Aizawa et al., 2012; Lang et al., 2012). HDAC9 has been found to express at a high level in the ischemic cerebral hemisphere of rats following cerebral ischemic/reperfusion injury (Shi et al., 2016). Moreover, as per our findings, ischemia led to an increase in the HDAC9 expression in the brain of wild type mice, while the loss of HDAC9 was observed to suppress the release of IL-1β, IL-6, IL-18, and TNF-α in the mouse cortex, hippocampus, and hypothalamus after I/R injury (Lu et al., 2018). In our research, it was found that HDAC9 was highly expressed in OGD-treated cells and that its depletion could repress neuron apoptosis as well as the release of inflammatory factors.

HDAC inhibitors have shown robust neuroprotection on cerebral ischemia-induced brain injury, which may be complex involving multiple mechanisms, such as the activated microglia-mediated inhibition of cerebral inflammation induced by ischemia (Kim et al., 2007). HDAC9 was found to be upregulated in OGD-induced brain microvessel endothelial cells (BMVECs), and its knockdown can attenuate the production of pro-inflammatory mediators in the BMVECs, thus preventing the progression of cerebral I/R injury (Shi et al., 2016). Additionally, HDAC9 has been reported to bind to and deacetylate IKKα and IKKβ, which subsequently led to their activation to modulate the inflammatory responses in the macrophages and endothelial cells. Besides, the pharmacological suppression of HDAC9 using a TMP195 inhibitor could limit the pro-inflammatory responses in macrophages (Asare et al., 2020). Consistent with those findings, deficiency of HDAC9 can abolish oxidized low-density lipoprotein-induced cell apoptosis and suppress the expression of oxidized low-density lipoprotein-induced inflammatory factors such as TNF-α and MCP1, thus retarding atherosclerosis development (Han et al., 2016). Thus, siRNA-mediated HDAC9 deficiency may exert neuroprotective effects against ischemic brain injury by reducing apoptosis and inflammatory responses.

We also found that HDAC9 suppressed the expression of miR-20a by enriching in its promoter region while silencing of HDAC9 promoted the expression of miR-20a. The inverse correlation between HDAC9 and miRNAs has been reported previously. For instance, ChIP assay in a previously conducted study revealed that the promoter region of miR-17-92a presents with HDAC9 enrichment in human periodontal ligament stromal cell samples (Li et al., 2018), suggestive of the inhibitory role of HDAC9 in the miR-17-92a expression by direct deacetylation. Glioma tissues and cells both present with a decreased expression of miR-20a-5p relative to the normal brain tissues and cells (Yang et al., 2018). Also, treatment with miR-20a mimic can abolish spinal cord injury-induced neuronal apoptosis (Liu et al., 2015). miR-20a upregulation is also shown to prevent proinflammatory cytokine secretion in macrophages by inhibiting its target gene SIRPα (Zhu et al., 2013). Transfection of miR-20a mimic reduced the release of IL-6, CXCL10, and IL-1β, as well as TNF-α by rheumatoid arthritis fibroblast-like synoviocytes (Philippe et al., 2013). These above-mentioned findings suggested the regulation of the HDAC9/miR-20a signaling in ischemic brain injury whereby HDAC9 silencing resulted in increased miR-20a expression, thus inhibiting OGD-induced neuronal apoptosis and inflammatory factor production.

Additionally, we found up-regulated expression of NeuroD1 in the MCAO-induced mouse model. An association has been demonstrated between several neurological disorders and severe neuronal loss, and the challenge of restoring lost neurons and impaired brain function has proven very difficult to solve. NeuroD1, as an endogenous neural transcription factor, has been implicated to express both in early brain development and in adult neural stem cells (Gao et al., 2009; Kuwabara et al., 2009). Unlike classical gene therapy that overexpresses a missing protein to treat a genetic defect, an alternative approach is to overexpress a neural transcription factor NeuroD1 and thus force differentiation of glial cells into new neurons (Chen et al., 2020). Expression of a particular NeuroD1 in the brain of monkeys can efficiently convert stress-induced astrocytes caused by ischemic brain injury into morphologically normal neurons (Chen et al., 2020). Moreover, as reported in a previous study (Ge et al., 2019), overexpression of the single transcription factor NeuroD1 in the brain of Alzheimer’s disease model mice can directly transform the stress-involved glial cells caused by brain injury into functional neurons *in situ*, which were further used for gene therapy to restore the brain function in the mouse model of cerebral ischemia. This finding suggests that NeuroD1 is an important factor that promotes neurogenesis. The relationship between NeuroD1 expression and neuronal gene induction was manifested by the present results that NeuroD1 directly binds either the promoters or enhancers of various upregulated genes. NeuroD1 has been reported as a critical regulator of neuronal development, during which it promotes neurogenesis and the migration of newborn neurons by directly modulating the underlying transcriptional program, which is beneficial for stroke recovery (Pataskar et al., 2016; Ranjan et al., 2020). Our results showed that miR-20a had a binding site in the 3′UTR of NeuroD1 mRNA and that miR-20a targeted NeuroD1 and down-regulated its expression, thus inhibiting the apoptosis of OGD-treated neurons. Partially consistent with our findings, miR-19b, which is a member of miR-17-92 cluster, could target the 3’UTR of NeuroD1 mRNA to decrease its protein and mRNA levels (Zhang et al., 2011). Bcl-2 is abundant in the nervous system during neural development and exerts a key role in the regulation of cell survival; suppressed NeuroD1 facilitates the neurite outgrowth induced by Bcl-2 overexpression (Lee et al., 2020), suggesting an inverse correlation between Bcl-2 and NeuroD1. Also, Neurod1 is a direct target gene of miR-101a and miR-30b, and the two miRs can mediate β-cell apoptosis by diminishing the expression of the anti-apoptotic protein Bcl-2 (Zheng et al., 2015). Also, NeuroD1 has the potency to antagonize miR-124-induced promoting effect on neuronal proliferation (Liu et al., 2011). Furthermore, miR-30a-5p ameliorates spinal cord injury-induced inflammatory responses by targeting NeuroD1 (Fu et al., 2018). Treatment of cultured neural precursor cells with an HDAC inhibitor elevates NeuroD1 expression (Jeon et al., 2014). In our study, depletion of HDAC9 by treatment with its specific shRNA was found to decrease the expression of NeuroD1 *in vitro* and *in vivo*. Thus, HDAC9 silencing might inhibit the apoptosis of OGD-treated neurons by regulating the miR-20a/NeuroD1 axis. Indeed, we found that decreased NeuroD1 expression attenuated the apoptosis of OGD neurons, which may be due to the initiation of transcription factor expression in the model, thus enhancing the occurrence of apoptosis. However, details of the interaction between HDAC9 and NeuroD1 remain unclear, which calls for further in-depth investigation.

In summary, our research demonstrated that silencing of HDAC9 could promote miR-20a expression, and then down-regulate NeuroD1 expression, consequently retarding ischemic brain injury progression. This finding provides insight to develop new therapeutic strategies based on HDAC9 targets for the prevention and treatment of ischemic brain injury. However, further effects of miR-20a/Neurod1 after HDAC9 regulation and other mechanisms of action of HDAC9 still need to be elucidated to establish the feasibility of HDAC9 as a therapeutic target for ischemic brain injury.

## Data Availability Statement

The original contributions presented in the study are included in the article/Supplementary Material, further inquiries can be directed to the corresponding author.

## Ethics Statement

The current study was approved by the Institutional Animal Use Committee of Shandong Provincial Hospital.

## Author Contributions

LZ and LM designed the study. JY and HL collated the data, carried out data analyses, and produced the initial draft of the manuscript. LZ and JY contributed to drafting the manuscript. All authors contributed to the article and approved the submitted version.

## Conflict of Interest

The authors declare that the research was conducted in the absence of any commercial or financial relationships that could be construed as a potential conflict of interest.

## Supplementary Material

SUPPLEMENTARY FIGURE 1 Expression of HDAC9 in different tissues and cells in the bioGPS website.

SUPPLEMENTARY FIGURE 2 Representative images of TUNEL staining. **(A)** Primary neuron apoptosis measured by TUNEL in cerebral tissues of sham-operated and MCAO mice. **(B)** Apoptosis of primary OGD-induced neurons treated with sh-HDAC9 measured by TUNEL. **(C)** Apoptosis of primary OGD-treated neurons treated with sh-HDAC9 or in combination with miR-20a inhibitor measured by TUNEL. **(D)** Apoptosis of primary OGD-treated neurons transfected with miR-20a mimic or in combination with oe-Neurod1 detected by TUNEL. **(E)** Apoptosis of primary OGD-treated neurons transfected with sh-HDAC9 or in combination with oe-Neurod1 detected by TUNEL.
