# Supplementary figures and images for: HDAC9 Silencing Exerts Neuroprotection Against Ischemic Brain Injury via miR-20a-Dependent Downregulation of NeuroD1
Source: Front Cell Neurosci. 2021 Jan 11;14:544285. doi: 10.3389/fncel.2020.544285 (PMC7873949; doi:10.3389/fncel.2020.544285)

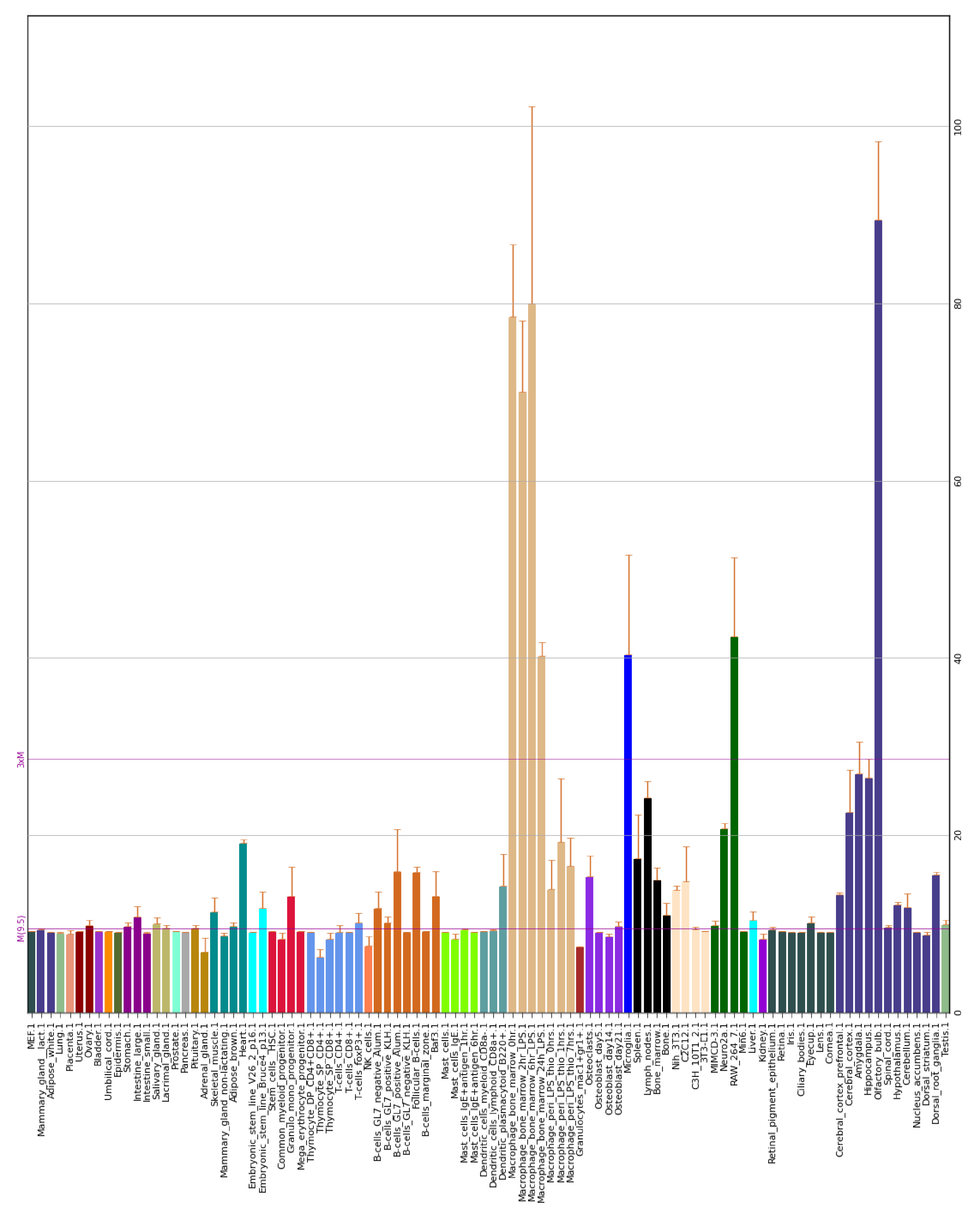

Supplement: SUPPLEMENTARY FIGURE 1 — Expression of HDAC9 in different tissues and cells in the bioGPS website. [file Image_1.JPEG]

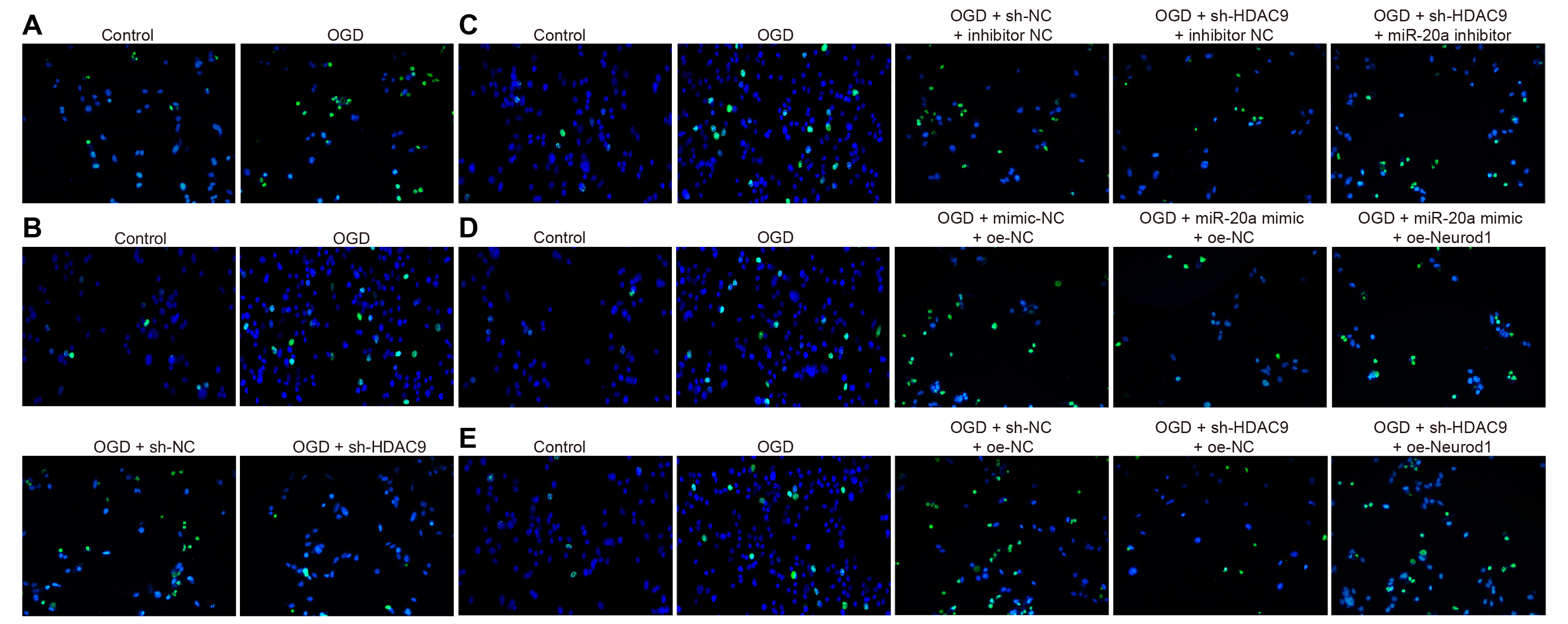

Supplement: SUPPLEMENTARY FIGURE 2 — Representative images of TUNEL staining. (A) Primary neuron apoptosis measured by TUNEL in cerebral tissues of sham-operated and MCAO mice. (B) Apoptosis of primary OGD-induced neurons treated with sh-HDAC9 measured by TUNEL. (C) Apoptosis of primary OGD-treated neurons treated with sh-HDAC9 or in combination with miR-20a inhibitor measured by TUNEL. (D) Apoptosis of primary OGD-treated neurons transfected with miR-20a mimic or in combination with oe-Neurod1 detected by TUNEL. (E) Apoptosis of primary OGD-treated neurons transfected with sh-HDAC9 or in combination with oe-Neurod1 detected by TUNEL. [file Image_2.JPEG]
